# Assessing Consumer Valuation of Sustainability Certification in Seafood Products: Insight from a Discrete Choice Experiment of Korean Blue Food Market

**DOI:** 10.3390/foods14162821

**Published:** 2025-08-14

**Authors:** Dong-Hun Go, Sangchoul Yi

**Affiliations:** 1 Korea Maritime Institute, Busan 49111, Republic of Korea; donghun.go@kmi.re.kr; 2 Department of Marine & Fisheries Business and Economics, Pukyong National University, Busan 48513, Republic of Korea

**Keywords:** Atlantic salmon, choice modeling, consumer preferences, discrete choice experiment, Korean seafood market, sustainable aquaculture certification

## Abstract

This study utilizes a discrete choice experiment (DCE) to estimate consumer valuation of sustainable aquaculture certification for Atlantic salmon (*Salmo salar*), one of the most important imported seafood products in South Korea. This experiment investigates consumer preferences across five product attributes: country of origin, product type, preparation method, sustainability certification, and price. Data were collected through an online survey administered by a professional research firm that yielded 24,000 valid choice observations from 1000 respondents. Conditional logit estimates revealed that all specified attributes significantly influenced consumer choices among seafood alternatives. Among the key product attributes, sustainability certification has emerged as the most influential factor affecting consumer decisions. The marginal willingness to pay for sustainably certified aquaculture is estimated to be USD 1.33 per 100 g. These findings provide valuable insights for seafood marketers and policymakers who seek to promote sustainable aquaculture in South Korea.

## 1. Introduction

The global aquaculture industry has experienced notable growth over the past several decades and emerged as a critical component of future food security systems [1,2]. This expansion has been driven by mounting pressure on wild fisheries and the urgent need to address escalating global protein demands [3]. According to the Food and Agriculture Organization, approximately 90% of the global marine fish stocks are either fully exploited or overexploited, with many commercially important species experiencing severe population decline [4,5,6]. Simultaneously, climate change, habitat destruction, and pollution have further compromised the productivity and resilience of natural fish populations [2,5].

Against the backdrop of declining wild fish availability, the global demand for seafood continues to rise dramatically. Population growth, urbanization, and increasing income levels in developing countries have created an unprecedented demand for high-quality protein sources [3]. The Food and Agriculture Organization (FAO) projects that global seafood consumption will increase by 12% between 2022 and 2032, requiring an additional 20.5 million tons of fish annually [7]. This growing demand-supply gap cannot be met by capture fisheries alone, creating an urgent need for alternative production methods [8].

Aquaculture has emerged as the most viable solution to bridge this protein gap, offering several distinct advantages over wild-capture fisheries [2,9]. Unlike capture fishing, aquaculture production can be scaled systematically to meet specific demand projections and provide predictable yields [10]. The controlled environment of aquaculture operations enables efficient feed conversion ratios, often producing more protein per unit of feed input compared to terrestrial livestock [3,11]. Furthermore, aquaculture can be established in diverse geographical locations including areas with limited agricultural potential, making it particularly valuable for food security in developing regions [11]. The sector’s potential to provide affordable nutrition has positioned itself as an essential pillar of sustainable food production strategies worldwide [4].

However, the rapid expansion of aquaculture operations has raised significant environmental concerns that cannot be overlooked [12]. The intensive nature of modern fish farming practices has generated substantial ecological impacts, including water pollution, habitat degradation, and disruption of local ecosystems [2,5]. These environmental externalities have prompted widespread calls for more sustainable approaches to aquaculture development, recognizing that the long-term viability of the industry critically depends on environmental stewardship [13].

The initial responses to these challenges relied primarily on regulatory approaches implemented at the national level [14]. While such government-led initiatives have achieved success in specific jurisdictions, their effectiveness remains fundamentally limited by the highly internationalized nature of seafood markets. Global trade networks that characterize the modern seafood industry mean that regulatory efforts in individual countries often fail to address the broad systemic issues inherent in aquaculture production chains [11].

Recognizing these limitations, international organizations have begun to develop comprehensive frameworks for sustainable aquaculture practices. The FAO has played a leading role in this effort by establishing guidelines for sustainable aquaculture development and promoting their adoption across member countries [15]. These initiatives reflect the growing international recognition of the need for coordinated approaches to sustainable aquaculture.

Complementing these institutional efforts, private sector initiatives have emerged as powerful drivers of sustainable aquaculture practices, particularly in European and American markets [1,16]. Market-based certification schemes for sustainable aquaculture have gained significant traction and offer alternatives to regulatory approaches. These certification systems, which are developed by industry associations and implemented by private certification bodies, provide sustainability credentials for aquaculture operations that meet predetermined environmental and social standards [17].

The appeal of these market-driven approaches lies in their ability to harness consumer preferences and market forces to incentivize sustainable practices. Instead of relying solely on regulatory compliance, certification schemes create economic incentives for sustainable production through enhanced market access and premium pricing opportunities [18]. For example, Vince and Haward [19] argued that third-party certification and private-social partnerships represent forms of hybrid governance that are transforming traditional modes of governance. By analyzing the case of ASC (Aquaculture Stewardship Council) certification in salmonid aquaculture in Tasmania, Australia, they illustrated how market and consumer engagement could challenge government policies. Such a market-based mechanism enables consumers to represent their preferences for environmentally friendly products, thereby creating direct economic incentives for sustainable aquaculture.

Despite growing industry adoption and consumer awareness of sustainable aquaculture certification, a critical knowledge gap remains regarding the economic value that consumers place on sustainability credentials [20]. Although environmental and consumer advocacy groups have successfully promoted certification schemes and expanded their market presence, the quantitative assessment of consumer valuation for sustainable aquaculture certification has received limited attention in the academic literature.

Previous research has attempted to address this gap through Contingent Valuation Methods (CVM), with several studies estimating the overall value that consumers place on sustainably produced seafood and certification labels [21,22,23]. However, the CVM approach presents methodological limitations in understanding the specific value attributable to individual sustainability attributes because it typically estimates the aggregate product value rather than decomposing the contribution of specific characteristics [24].

To address these methodological limitations, this study employs a discrete choice experiment (DCE) approach to estimate the consumer valuation of sustainable certification for Atlantic salmon. While DCE models have been frequently used in the fields of general food and seafood research [25,26,27], studies specifically measuring the value of sustainable seafood remain limited [28]. Previous studies have primarily focused on attributes such as country of origin or product characteristics [29]. The DCE methodology offers significant advantages over traditional CVM approaches by enabling the disaggregation of product attributes and providing more precise estimates of the value that consumers assign to specific sustainability characteristics.

**Figure A1:**
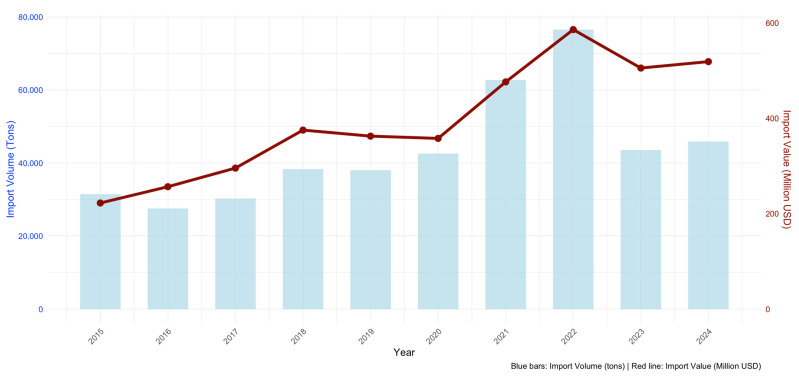
Atlantic Salmon Import Volume and Value Trends.

**Figure A2:**
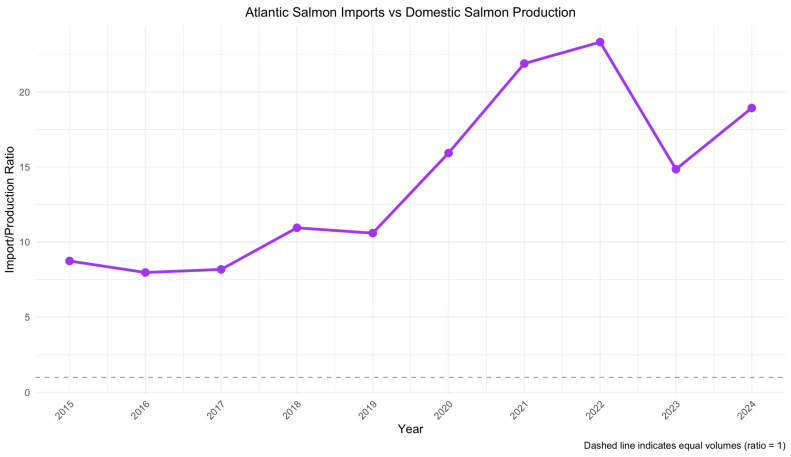
Import to Domestic Production Ratio.

The Republic of Korea (South Korea) is one of the major importers of seafood products in the global seafood market. The country is experiencing a rapid increase in the consumption of Atlantic salmon (see Figure A1 and Figure A2). As a key consumer of Atlantic salmon in the Asian region, South Korea could serve as an informative case study for understanding Asian consumers’ characteristics. However, the characteristics of seafood consumption in the Korean market differ significantly from those in Western countries. In terms of dietary habits, a large proportion of seafood is consumed in raw form [30], with freshness being a critical factor. Similarly, most salmon is consumed as a raw product in Korea, primarily for sushi. In contrast, Western markets typically favor value-added salmon products, such as smoked salmon and filets for salmon steaks.

This study contributes to both academic knowledge and practical policy development by providing quantitative estimates of the consumer valuation of sustainable Atlantic salmon production. These findings could inform decision-making regarding the future direction of domestic Atlantic salmon aquaculture development while advancing the broader literature on consumer preferences for sustainable seafood products. By quantifying the economic value of sustainability efforts in salmon aquaculture, this study offers valuable insights for industry stakeholders, policymakers and researchers working to promote environmentally responsible aquaculture practices.

## 2. Methods

### 2.1. Survey Design and Implementation

This study employs a DCE model to estimate consumers’ willingness to pay for sustainable seafood. Estimating the DCE model requires the development of a DCE survey instrument and a well-structured questionnaire design. The researchers identified key explanatory variables with suitable levels through market research and a review of prior studies [31,32,33] and incorporated them into the survey instrument.

**Figure 1:**
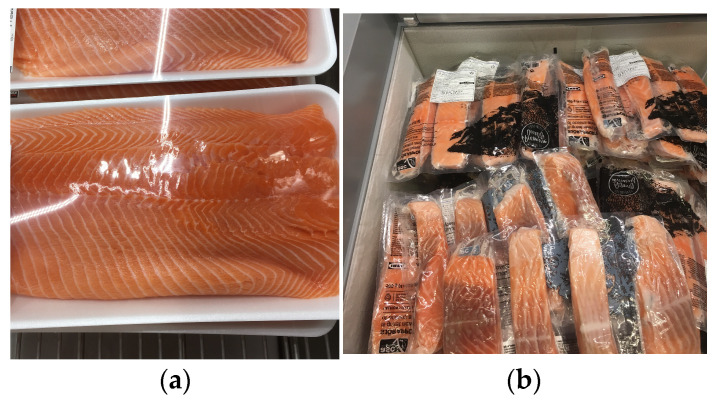
Visual representation of choice alternatives across product types: (**a**) fresh salmon; (**b**) frozen salmon.

One of the primary explanatory attributes is the Country of Origin (COO). To construct the levels of this attribute, Norway and Chile were selected as the major exporters of salmon imported to the Korean market, along with domestically produced salmon as a potential alternative. Given that salmon is distributed as both freshly chilled and frozen products in the Korean market, this distinction was included in the survey instrument (Figure 1).

Additionally, based on consumption purposes, the processed status of salmon, such as sashimi-grade, cooked, or smoked salmon, was included as a sub-level. Another critical attribute, sustainability certification, for aquaculture products is categorized into certified and non-certified products. Five price levels were established based on recent price trends in the domestic market. A summary of these attributes and their levels is presented in Table 1. Initially, a total of 180 alternative combinations (2 × 3 × 3 × 2 × 5) were generated. However, to efficiently implement experimental designs for discrete choice experiments, this study employed specialized software, Sawtooth Software CBC version 5. The Sawtooth module draws from a subset of the full choice design for each respondent while ensuring level balance and near-orthogonality within individual profiles [34]. From this process, 55 unique combinations were selected and used in the final design.

Prior to the main online survey, a pre-test was conducted with five research team members, who evaluated the questionnaire from a respondent’s perspective. The main data collection was carried out through an online survey administered by a professional research firm, which operates the largest online survey panel in South Korea. The survey sample was designed to ensure demographic representativeness through stratified sampling. The sampling framework and the survey implementation considered demographic characteristics such as gender and age distributions in South Korea.

In total, 24,000 valid responses were obtained from 1000 respondents. However, after excluding 2134 responses in which the respondents selected the opt-out option, indicating that they chose neither of the two alternatives, a total of 21,866 responses remained for analysis.

### 2.2. Discrete Choice Experiment Framework

This study used a DCE to evaluate consumers’ stated preferences and willingness to pay (WTP) for Atlantic Salmon products. The choice experiment is based on Random Utility Maximization (RUM) theory [35] and McFadden’s choice model [36]. Following the RUM theory, the utility function that individual (consumer) i gains from alternative j in the choice set can be expressed as Equation (1):

(1) $$U_{ij}=V_{ij}+\varepsilon_{ij}$$

where $V_{ij}$ refers to the deterministic part of utility that is observable and gained from alternative j chosen by consumer i. On the other hand, $\varepsilon_{ij}$ refers to the error component, or a random part of utility that is not observable. Following Jumamyradov et al. [37], alternative j is chosen by consumer i if and only if

(2) $$P_{ij}=P\left(U_{\mathrm{ij}}>U_{\mathrm{ik}},\;\forall k\neq j\right)=P\left(V_{ij}+\varepsilon_{ij}>V_{ik}+\varepsilon_{ik}\right)=P\left(V_{ij}-V_{ik}>\varepsilon_{ik}-\varepsilon_{ij}\right)$$

Under the assumption of the conditional logit (CL) model that the error term $\varepsilon_{ij}$ is independently and identically distributed (IID), and follows a Type I extreme value distribution [36], the term $\varepsilon_{ik}-\varepsilon_{ij}$ is also logistically distributed. Thus, the probability of consumer i selecting alternative j is expressed by using the conditional logit model as follows:

(3) $$P_{ij}=\frac{1}{1+\sum_{k\in C_{i}}exp(V_{ik}-V_{ij})},\;\forall k\neq j$$

In this study, respondents were required to choose among three alternatives in a choice experiment. They were asked to choose one alternative considering the trade-offs among the attributes of ‘country of origin’, ‘product type’, ‘preparation methods’, ‘sustainability certification’, and ‘price’.

The choice result of alternative j selected by consumer i is denoted by the indicator function $A_{ij}=1(\cdot )$. Then, $A_{ij}$ has a value of one when the j-order alternative is chosen by i-order consumer, and zero otherwise.

However, the specific functional form of $V_{ij}$ is assumed to be linear [38], as shown in Equation (4) below:

(4) $$V_{ij}=\sum_{m=1}^{M}\beta_{m}X_{ijm}$$

where $\beta_{m}$ represents the coefficient of estimation for the attribute, and $X_{ijm}$ the attribute presented in the survey, which includes ‘country of origin’, ‘product type’, ‘preparation methods’, ‘sustainability certification’, and ‘price’, as mentioned before.

From Equation (4), the marginal willingness to pay (MWTP) can be derived by setting a differential equation for each attribute [39]. MWTP represents the change in consumers’ marginal utility, expressed as a standardized value in monetary units [38]. For example, based on Equation (4), consider the price attribute p for consumer i choosing alternative j as $X_{ijp}$, and the remaining attributes as $X_{ijm},\;\forall p\neq m$. In this case, the conditional logit model estimate was calculated as follows:

(5) $$MWTP=\frac{dV/dX_{m}}{dV/dX_{p}}=-\frac{\beta_{m}}{\beta_{p}},\;\forall p\neq m$$

## 3. Results and Discussion

### 3.1. Sample Characteristics

The demographic characteristics of the participants are summarized in Table 2. The sample demonstrated a balanced sex distribution with 51.1% (*n* = 511) men and 48.9% (*n* = 489) women.

Participants were relatively evenly distributed across age groups, with the largest proportion in their 50s (23.5%, *n* = 235), followed by those in their 40s (22.2%, *n* = 222), 30s (18.8%, *n* = 188), and 20s (18.3%, *n* = 183). Participants aged 60 and above constituted 17.2% (*n* = 172) of the sample. Regarding marital status, the majority of participants were married (64.6%, *n* = 646), while unmarried individuals accounted for 35.4% (*n* = 354). The monthly income distribution showed considerable variation across the seven income brackets, with the highest proportion earning between $4303 and $5741 (17.5%, *n* = 175) and those earning $5741 or more (17.4%, *n* = 174). The middle-income categories of $2877–$3590 and $2154–$2877 represented 16.3% (*n* = 163) and 14.9% (*n* = 149), respectively, while the $1441–$2154 and $3590–$4303 brackets each comprised 13.6% (*n* = 136). Notably, only 6.7% (*n* = 67) of participants reported monthly income of less than $1441. The demographic profile indicated a predominantly middle-to upper-middle-class sample with balanced gender representation and a broad age distribution.

### 3.2. Estimation Results

Table 3 presents the conditional logit model results for seafood product choice, demonstrating strong overall model significance (Wald χ^2^(7) = 1406.25, *p* < 0.001; Pseudo R^2^ = 0.182). The results revealed that sustainability certification had a substantial impact on consumer choice and price premiums, with a coefficient of 0.9010 (*p* < 0.001), indicating a strong preference for sustainable seafood options over unsustainable alternatives. A form of product consumption also emerged as a critical factor, with fresh salmon significantly preferred over frozen products (β = 0.6465, *p* < 0.001). Among food preparation, sushi preparation showed a highly significant positive effect (β = 0.5814, *p* < 0.001) compared to smoked salmon, while grilled preparation demonstrated a marginally significant preference (β = 0.0879, *p* = 0.070). These results suggest that salmon was introduced and consumed in South Korea as a premium ingredient in sushi. Given that sushi is generally regarded as a premium food product, a price premium is likely to exist for fresh salmon on the market.

Country of origin effects revealed significant consumer preferences, with Korean seafood showing a positive preference (β = 0.4374, *p* < 0.001) and Norwegian seafood also preferred (β = 0.2010, *p* < 0.001) relative to Chilean products. As anticipated, price sensitivity was confirmed through a significant negative coefficient (β = −0.0005, *p* < 0.001), indicating that higher prices reduce the probability of product selection. These results are consistent with the fundamental economic principles of consumer demand behavior. Based on these findings, we can estimate the price premium for each attribute to better understand consumers’ willingness to pay for specific product characteristics.

Based on the results of the conditional logit model, Table 4 translates the estimated coefficients into marginal WTP values, providing monetary measures of consumer preference for each salmon attribute. These WTP estimates are calculated using the concept of price sensitivity, specifically by taking the negative ratio of each attribute coefficient to the price coefficient, which reveals the price premiums consumers are willing to pay for specific product characteristics. The sustainability premium emerged as the most substantial, with consumers willing to pay an additional 1850.50 KRW (1.33 USD) per 100 g for sustainable salmon. This finding directly reflects the strong positive coefficient (β = 0.9010) observed in the choice model, confirming that environmental certification represents the most influential non-price factor in consumer decision-making.

Fresh salmon commands the second-highest premium at 1327.85 KRW (0.96 USD) over frozen alternatives, corresponding to a significant positive coefficient (β = 0.6465) in the choice model. This substantial premium emphasized the importance of product freshness in the Korean salmon market. The premium for the sushi-grade product is 1194.15 KRW (0.86 USD), reflecting a highly significant coefficient (β = 0.5814). This value quantifies how salmon’s association with premium sushi culture translates into tangible price premiums.

The country-of-origin premiums reveal interesting patterns, with Korean-origin salmon commanding 898.38 KRW (0.65 USD) more than Chilean salmon, whereas Norwegian salmon has a premium of 412.77 KRW (0.30 USD). These monetary values directly correspond to the positive coefficients for Korean (β = 0.4374) and Norwegian (β = 0.2010) origins identified in the choice model. These marginal WTP estimates offer actionable insights for market participants, demonstrating that consumers are willing to pay substantial premiums for attributes associated with quality, sustainability, and preferred origin. This cumulative effect may justify significant price differentiation in the marketplace.

**Figure 2:**
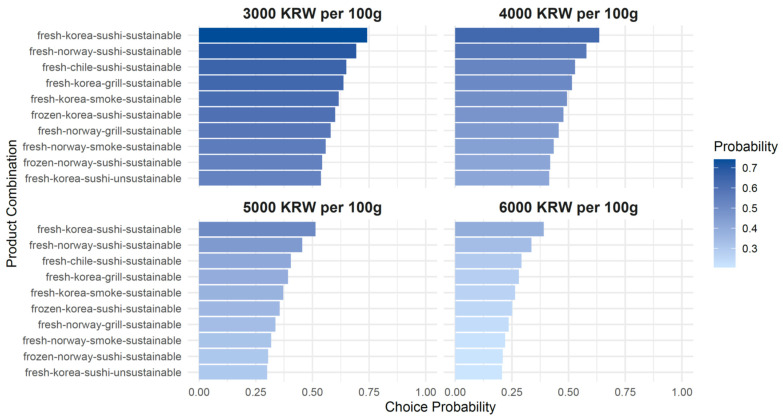
Product Choice Probability by Price Level (Top 10 Combinations).

In addition to estimating MWTP using the results of the Conditional Logit Model, the estimation outcomes can also be applied in the field of marketing. At each standardized unit price level, the choice probabilities associated with different product attribute combinations can be computed. These probabilities are visualized in Figure 2, which illustrates how consumers are likely to choose among products with varying attribute bundles when presented at the same unit price. These results provide practical insights for food marketers by offering valuable guidance for product planning, pricing strategies, and feature configuration decisions in the context of market positioning and sales.

Table 5 presents the results of the conditional logit model for seafood choice across the three income levels. Income is a key socioeconomic variable that significantly influences various aspects of food consumption, including purchasing power, preferences, access to information, and risk attitudes [40]. To analyze how preferences vary by income level, this study segmented the sample into high-, middle-, and low-income groups. The conditional logit model is suitable for analyzing the effects of alternative-specific independent variables. However, it has limitations in directly capturing the influence of case-specific variables, such as income level. To statistically examine differences across groups based on income level, interaction terms were introduced, and model comparisons were conducted. Specifically, a likelihood ratio test was performed to compare a full model including interaction terms for income level with a reduced model excluding them. The results confirmed significant differences in coefficients across income groups (LR χ^2^(14) = 44.07, *p* < 0.0001).

Product freshness significantly influences consumer choice across all income levels. Regarding the country of origin, Korean products were significantly preferred over Chilean products (reference category) in every income group, with the strongest preference observed among high-income consumers. Norwegian products showed significant positive effects for both the low- and high-income groups, but the results were not statistically significant for middle-income consumers.

Regarding preparation methods, sushi was significantly preferred over smoked products (the reference category) across all income levels, with coefficients rising from 0.571 for low-income consumers to 0.796 for high-income consumers. Grilled preparations showed no significant effects in any income group. Sustainability emerged as the most influential attribute, with all income groups displaying strong preferences for sustainable products; the middle-income group recorded the highest sustainability coefficient (β = 1.067), followed by high-income (β = 0.790) and low-income (β = 0.794). The price coefficients were negative and statistically significant for the low- and middle-income groups, but not for the high-income group, indicating that only the former two segments exhibited apparent price sensitivity. Although the differences in income levels were not statistically significant, these findings align with the economic theory. The absence of a statistical significance for the high-income group suggests that consumers in this segment enjoy greater economic freedom, thus constructing a premium market segment that is less sensitive to price changes.

Studies on sustainably produced seafood have been conducted in various markets. To date, research has proceeded mainly in two directions. One stream focuses WTP estimation studies for products using the CVM. The other examines marginal WTP estimations for individual product attributes using DCE methods. This study employed the DCE approach to estimate consumer preferences and values regarding seafood attributes such as sustainability certification and demonstrated statistically significant results. These findings are consistent with those of previous studies.

Bronnmann et al. conducted research on salmon products in the German seafood market, estimating the value of sustainably produced salmon, and argued that sustainability information positively influences consumer choice [41]. Similarly, James et al. analyzed point-of-sale scanner data from a regional upscale US supermarket and found that sustainability information positively affects consumer choice and product valuation [42]. Davide et al. applied DCE models to investigate consumer demand and choice behavior for fish products across five European countries (France, Germany, Italy, Spain, and the United Kingdom) to evaluate consumer preferences and WTP for sustainability labels. Consistent with the findings of this study, their research demonstrates that consumers are willing to pay price premiums for fish products with sustainability labels. However, consumer preferences vary significantly by country and fish species [21].

Among the product attributes that influence consumer choice, a noteworthy factor is the effect of COO. In Korea, most salmon products are imported, and domestic salmon production is promoted to address this situation. In this context, estimating the price premium that domestically produced salmon can command is significant. According to the results, the country of origin of products has a substantial impact on consumer product choice, with a particularly high preference for domestically produced products. These findings align with the results reported by Onozaka et al., who investigated consumer preferences when domestic salmon was produced in markets in France, Japan, and the United States [29]. However, their study compared only domestic and Norwegian salmon species. This study identified the COO effect on domestic salmon currently under production development alongside Norwegian and Chilean salmon, which currently dominate the Korean market. Banović et al. conducted analysis using categories similar to those established in this study, dividing COO label information into categories such as “Produced in the EU” and “Produced in own country”. They found that COO and ASC labels were more influential than health claims in driving consumer preferences [43].

This study focuses on product distribution conditions. Two types of product distributions were considered: fresh and frozen. These distribution categories significantly impact the Atlantic salmon market. For instance, there are differences in freshness and taste, and distribution costs vary greatly, ultimately resulting in substantial price differences. Fresh salmon products are imported by air transport, resulting in higher distribution costs than actual production costs.

In contrast, frozen products are transported by ship, offering advantages in terms of distribution costs and longer product shelf life. This study showed that general consumers demonstrated a greater preference for fresh and chilled products. These results are similar to those found by Ankamah-Yeboah et al., who indicated that fresh salmon products command an additional price premium of approximately 30% compared with frozen products [44]. Ahmad et al. conducted a consumer preference analysis across marine fish, not limited to farmed seafood, and reported that fresh seafood commands higher price premiums than frozen products [45].

Product consumption format is a crucial variable that defines the Atlantic salmon market in Korea. Unlike Western countries, Korea has a substantial demand for Atlantic salmon as a sushi ingredient, and this study revealed an even higher preference and price premium compared with the country of origin. While previous studies often compared fried filets or smoked product categories [31,46,47], this study provides a more detailed segmentation of the product consumption format, presenting price premiums for sushi-use products.

## 4. Conclusions

In this study, we conducted a discrete choice experiment to investigate consumer preferences for Atlantic salmon based on five product attributes: country of origin, product type, preparation method, sustainability certification, and price. Data were collected via an online survey administered by a professional research firm in Korea, which yielded 24,000 valid choice observations from 1000 respondents. Conditional logit estimates revealed that each of the specified attributes significantly influenced consumers’ choices of seafood alternatives.

This study makes several significant contributions to the literature on seafood consumption and sustainability. First, by focusing on Atlantic salmon, a globally representative traded seafood product, we analyze the responses of consumers in South Korea, a primary salmon consumption market. This approach provides a crucial reference point for international comparative studies and offers insights into key consumer markets. Thus, this study established an internationally comparable benchmark.

Second, employing a DCE, we estimated the marginal willingness to pay for sustainable aquaculture certification. In doing so, we quantified the economic value that consumers assigned to sustainability efforts in the aquaculture sector, providing important implications for industry practices and policies.

This study has some limitations. For an international comparative analysis, this study focused on a single species, Atlantic salmon, which is a widely traded species. However, the price premium, which is a key factor in sustainable aquaculture, could vary depending on the species. Therefore, future studies should include additional research on other representative aquaculture species in Korea, such as the olive flounder, in addition to Atlantic salmon. In addition, this study focuses on sustainable aquaculture certification in its general sense. However, various types of sustainable aquaculture certifications such as ASC, organic certification, and global G.A.P. are being introduced into the aquaculture sector. Future research should examine sustainable aquaculture certification in a more specialized manner. Finally, due to the nature of the study, a conditional logit model was employed to estimate the marginal values of each attribute. However, this modeling approach is only appropriate for analyzing the effects of alternative-specific independent variables. Future research could apply models such as the Latent Class Model (LCM) or Random Parameters Logit (RPL) to gain deeper insights into the influence of possible socio-demographic factors.

## Figures and Tables

**Table 1:** Conjoint Choice Experiment Attributes and Levels.

Attribute	Levels
Product type	1. Fresh, 2. Frozen
Country of origin	1. Korea, 2. Norway, 3. Chile
Preparation methods	1. Sushi, 2. Grilled, 3. Smoked
Sustainability certification	1. Sustainable, 2. Unsustainable
Price	Five levels of prices based on the market conditions:

Notes: Reference categories are ‘Frozen’ (product type), ‘Chile’ (country of origin), ‘Smoked’ (Preparation methods), and ‘Unsustainable’ (sustainability certification).

**Table 2:** Demographic information of survey participants and reference population.

Research Samples				Reference Population Characteristics *			
Variable	Category	*n*	%	Variable	Category	*n*	%
Gender	Female	489	48.9	Gender	Female	25,846,661	49.99%
	Male	511	51.1		Male	25,837,903	50.01%
Age	20s	183	18.3	Age	20s	6,136,764	13.16%
	30s	188	18.8		30s	6,975,568	14.96%
	40s	222	22.2		40s	7,717,016	16.55%
	50s	235	23.5		50s	8,660,370	18.57%
	60 and above	172	17.2		60 and above	17,147,056	36.77%
Marital Status	Married	646	64.6	Marital Status	Married	30,269,089	68.9%
	Unmarried	354	35.4		Unmarried	13,688,458	31.1%
Monthly Income	Less than $1441	67	6.7	Average monthly income		$3757	-
	$1441–$2154	136	13.6				
	$2154–$2877	149	14.9				
	$2877–$3590	163	16.3				
	$3590–$4303	136	13.6				
	$4303–$5741	175	17.5				
	$5741 or more	174	17.4				

Note: currency converted using an exchange rate of USD 1 = KRW 1388; * Statistics Korea, “Household Income and Expenditure Survey”, 2024 Q4.

**Table 3:** Conditional Logit Model Results for Seafood Product Choice.

Variables	Coef.	Robust SE	z	*p*-Value	95% Conf. Interval	
**Product type (ref. Frozen)**						
Fresh ***	0.6465	0.0317	20.41	0.000	0.584	0.709
**Country of origin (ref. Chile)**						
Korea ***	0.4374	0.0813	5.38	0.000	0.278	0.597
Norway ***	0.2010	0.0450	4.46	0.000	0.113	0.289
**Preparation (ref. Smoked)**						
Sushi ***	0.5814	0.0627	9.28	0.000	0.459	0.704
Grilled	0.0879	0.0486	1.81	0.070	−0.007	0.183
**Sustainability (ref. Unsustainable)**						
Sustainable ***	0.9010	0.0595	15.13	0.000	0.784	1.018
Price ***	−0.0005	0.0001	−4.01	0.000	−0.001	−0.000
Constant	−0.0142	0.0230	−0.62	0.536	−0.059	0.031

Note: The dependent variable was seafood product choice. Number of observations: 21,826. Number of cases: 10,913. Log pseudolikelihood: −6187.231. Wald χ^2^(7): 1406.25 ***. Pseudo R^2^: 0.182. * *p* < 0.05, ** *p* < 0.01, *** *p* < 0.001. Marginal WTP is the extra amount that consumers pay per 100 g of each attribute versus the reference.

**Table 4:** Summary of Marginal Willingness to Pay by Product Attribute.

Product Attribute	Reference Category	Marginal WTP (KRW)	Marginal WTP (USD)
Sustainable	Unsustainable	1850.50	1.33
Fresh	Frozen	1327.85	0.96
Sushi	Smoked	1194.15	0.86
Korea origin	Chile	898.38	0.65
Norway origin	Chile	412.77	0.30
Grilled	Smoked	180.55	0.13

Notes: Marginal WTP represents the additional amount that consumers are willing to pay per 100 g for each attribute relative to the reference category, based on an exchange rate of USD 1 = KRW 1388.

**Table 5:** Conditional Logit Model Results by Income Level.

Product Attribute	Income Level		
	**Low**	**Middle**	**High**
**Product type (ref. Frozen)**			
Fresh	0.6070 ***	0.6620 ***	0.6950 ***
	(0.0555)	(0.0500)	(0.0607)
**Country of origin (ref. Chile)**			
Korea	0.4720 ***	0.2920 *	0.6360 ***
	(0.1350)	(0.1340)	(0.1550)
Norway	0.2340 **	0.1080	0.3090 ***
	(0.0794)	(0.0719)	(0.0817)
**Preparation (ref. Smoked)**			
Sushi	0.5710 ***	0.4700 ***	0.7960 ***
	(0.0970)	(0.1080)	(0.1240)
Grilled	0.0463	0.0582	0.2020 *
	(0.0801)	(0.0830)	(0.0900)
**Sustainability (ref. Unsustainable)**			
Sustainable	0.7940 ***	1.0670 ***	0.7900 ***
	(0.0957)	(0.0967)	(0.1210)
**Price**	−0.0006 **	−0.0007 ***	−0.0000
	(0.0002)	(0.0002)	(0.0002)
Constant	−0.0180	−0.0606	0.0170
	(0.0395)	(0.0357)	(0.0469)
**Model fit statistics**			
Observations	7630	8702	5494
Cases	3815	4351	2747
Log pseudolikelihood	−2194.72	−2425.79	−1544.65
Wald χ^2^(7)	519.81 ***	603.33 ***	331.92 ***
Pseudo R^2^	0.170	0.196	0.188

Notes: The coefficients with robust standard errors are shown in parentheses. The dependent variable was the seafood product choice. * *p* < 0.05, ** *p* < 0.01, *** *p* < 0.001.

## Data Availability

The original contributions presented in the study are included in the article, further inquiries can be directed to the corresponding author.

## Abbreviations

The following abbreviations are used in this manuscript:

ASC	Aquaculture Stewardship Council
CVM	Contingent Valuation Methods
COO	Country of Origin
DCE	Discrete Choice Experiment
FAO	Food and Agriculture Organization
IID	Independently and Identically Distributed
MWTP	Marginal Willingness to Pay
